# Teaching Health Insurance and Patient Barriers Through an Interprofessional Escape Room for Preclinical Medical and Health Professional Students

**DOI:** 10.15766/mep_2374-8265.11647

**Published:** 2026-10-30

**Authors:** Kriti Devkota, Rachel Schlak, Lia Spencer, Sar Kass, Khuram Ghumman, Douglas McHugh

**Affiliations:** 1 Medical Student, Department of Medicine, Frank H. Netter MD School of Medicine, Quinnipiac University; 2 Medical Student, Department of Medicine, Frank H. Netter MD School of Medicine, Quinnipiac University; 3 Medical Student, Department of Medicine, Frank H. Netter MD School of Medicine, Quinnipiac University; 4 Graduate Student, Department of Social Work, Quinnipiac University; 5 Director of the Primary Care Clerkship, Department of Medicine, Frank H. Netter MD School of Medicine, Quinnipiac University; 6 Assistant Dean for the Foundational Science Curriculum, Department of Medicine, Frank H. Netter MD School of Medicine, Quinnipiac University

**Keywords:** Health Insurance, Interprofessional Education, Escape Room, Patient Barriers, Games, Health Systems, Case-Based Learning

## Abstract

**Introduction:**

Health insurance is a crucial component of patient care, yet current medical education lacks adequate formal training in this subject matter. Escape rooms, a novel educational modality, have been shown to strengthen knowledge across diverse medical topics but have not yet been used in the context of health insurance education.

**Methods:**

We created a 2-part event that combined an informative 1-hour lecture session with 3 interactive patient case-based escape rooms to navigate the complexities of health insurance. Preclinical first- and second-year medical students and other health professional students completed a preevent survey to assess baseline knowledge and self-efficacy, with follow-up surveys administered 4 months post event to assess long-term retention of knowledge and self-efficacy.

**Results:**

Twenty-nine preclinical medical and health professional students participated. The intervention was feasible and well received by learners, with 100% of respondents reporting opportunities for interprofessional teamwork and problem solving, and 93% noting improved understanding of other health professionals’ roles. At 4 months, all participants reported significantly greater self-efficacy in their ability to address health insurance-related challenges (*MD* = 6; *SD* = 3.67; *P* < .01), though knowledge scores did not significantly improve from baseline (*MD* = 1.17; *SD* = 2.38; *P* = .06).

**Discussion:**

This pilot intervention demonstrates the feasibility of using escape rooms for health insurance education and highlights the modality's potential to promote teamwork and learner confidence. Future iterations should include spaced repetition of key concepts and longitudinal reinforcement to optimize knowledge retention. Additional research is warranted to evaluate the impacts on physician practice and patient outcomes.

## Educational Objectives

By the end of this activity, learners will be able to:

1. Identify different types of public health insurance plans (Medicare and Medicaid) and types of private health insurance networks (health maintenance organizations [HMOs], preferred provider organizations [PPOs], etc.), as demonstrated on knowledge assessment surveys.
2. Develop confidence in navigating health insurance concepts as a health care provider, as measured by self-efficacy surveys.
3. Demonstrate interprofessional collaboration to solve simulated patient case-based escape rooms.
4. Apply knowledge of barriers to patient care and facilitate access to relevant community resources and referral pathways in simulated patient case-based escape rooms.

## Introduction

Navigating health insurance systems is a challenge across the U.S. health care landscape, affecting both patients and the clinicians expected to guide them. Health insurance is a ubiquitous component of medical practice, yet medical students often report limited understanding of health insurance plans and difficulty assisting patients with the burden of health care costs.^1^ Notably, 86% of medical students feel a professional responsibility to engage with health policy,^2^ yet medical schools provide an average of only 14 hours of health policy education over 4 years,^3^ underscoring the need for more comprehensive training in this area.

Health insurance literacy is especially challenging because it requires learners to integrate terminology, structures, rules, and financial concepts into a logical mental framework. This integration is confusing for many people, regardless of education level.^4^ Previous case-based learning exercises among medical students have demonstrated improved knowledge of topics such as Medicare, Medicaid, and the Affordable Care Act,^5,6^ as well as increased self-perceived readiness to identify and address barriers to patient care in future medical practice.^5^ Patient health literacy is also associated with greater engagement with preventative care,^7^ suggesting that physicians with a comprehensive understanding of health insurance are better equipped to educate their patients and deliver patient-centered care.

The core problem addressed by this educational event is the insufficient health insurance education in medical training, which limits physicians’ ability to support patients as they navigate costs and coverage. To address this gap, we designed a two-part educational event consisting of an informative lecture and an interactive escape room. Escape rooms are learner-centered activities that encourage critical thinking and collaboration to solve problems to “escape” the room. They represent a novel trend in medical education that is engaging and effectively increases students’ knowledge across diverse topics.^8-10^ Prior medical education escape rooms have engaged teams of students in simulated scenarios, sequential puzzles, and decision-making organized around a learning theme.^11^ In our design, the lecture introduced foundational concepts that were then intentionally embedded into the escape room puzzles to promote active retrieval and applied reasoning.

This applied format naturally lends itself to team-based learning, creating an ideal environment to practice the interprofessional competencies that underscore effective patient-centered care. Interdisciplinary collaboration involves integrating the perspectives and methods of multiple areas of health care practice to develop shared solutions. Interprofessional collaboration expands this concept by further emphasizing holistic, team-based care that centers specifically around the patient.^12^

Guided by Interprofessional Education (IPE) competencies and experiential learning theory, our educational event is the first to utilize the escape room modality for teaching medical students about health insurance. Pairing a lecture with the escape room allowed us to first teach the fundamentals of health insurance to the attendees, then reinforce that knowledge through application in interprofessional teams. By simulating health insurance-related puzzles in an interprofessional environment, we aimed to foster communication, collaboration, and creative problem-solving skills essential for navigating the real-world complexities of insurance. To our knowledge, this is the first escape room educational event to reinforce health insurance knowledge through structured interprofessional collaboration.

## Methods

### Curricular Context and Target Learners

We implemented this educational event through Quinnipiac University's Center for Interprofessional Healthcare Education as an optional program for preclinical medical students (first- and second-year students) and health professional students from other disciplines, including nursing, social work, occupational therapy, and physical therapy. The event was part of the center's ongoing interprofessional programming designed to supplement existing curricular requirements. Students did not require any prerequisite knowledge, qualifications, or training to participate. Ethical approval was provided by the Institutional Review Board (IRB) at Quinnipiac University (IRB approval #02425), and participants provided written informed consent for event participation and survey data collection.

### Speakers and Lecture Preparation

Speakers included a board-certified family medicine physician and a master of social work graduate student. Speakers were briefed on the learners’ education level and escape room content in advance to ensure appropriateness of lecture topics.

### Lecture Component Design and Implementation

Prior to the event, students were provided with optional prework consisting of videos and articles highlighting patient perspectives on challenges with health insurance (Appendix A). This prework was designed to maximize appreciation of impactful firsthand experiences with health insurance but was designated optional to maximize participation by students. The event took place on April 3, 2025, at Quinnipiac University. The program began with a 1-hour lecture delivered by the aforementioned speakers, followed by a 30-minute question-and-answer session.

The physician speaker covered topics such as the Affordable Care Act, Medicare, Medicaid, private insurance network types (eg, health maintenance organizations [HMOs], preferred provider organizations [PPOs], and point of service), and common health insurance terminology (eg, premium, deductible, copay, out-of-pocket maximum, in-network, and dependent). The social work speaker covered specific eligibility criteria and coverage for components of Connecticut's Medicaid program, HUSKY, in addition to various social welfare programs, including the Supplemental Nutrition Assistance Program (SNAP). Presentation slides and speaker notes are included in Appendix B.

### Interactive Component Design and Implementation

Following the lecture, students participated in 3 interprofessional escape rooms at the Quinnipiac University Standardized Patient and Assessment Center, applying their knowledge through collaborative problem solving in case-based scenarios. To ensure a cohesive instructional design, each escape room was deliberately mapped to specific concepts introduced during the lecture. Facilitators were trained to prompt learners to recall and apply relevant lecture material during the escape room, thereby reinforcing structured retrieval throughout the activity.

The facilitators piloted the escape room with 5 first-year medical students (not included in the study population) to identify and resolve implementation issues prior to event day. Two escape rooms were designed to simulate realistic and common patient encounters: one focused on a pregnant patient eligible for Medicaid requiring social services and genetic counseling, and another focused on a Medicare patient requiring hip replacement due to osteoarthritis unresponsive to conservative management. Both cases were developed in accordance with Connecticut's HUSKY program^13^ and Medicare regulations^14^ to ensure fidelity to real-world processes. A third escape room required students to apply their knowledge of insurance terms and definitions in a matching game, which led to a break room. Completing the escape rooms involved interdisciplinary collaboration, correctly applying knowledge of health insurance to solve jigsaw and matching puzzles, and acknowledging and overcoming barriers to patient care with community resources. On event day, students were randomly assigned to interdisciplinary groups of 4-5 people using a random number generator and given up to 20 minutes to complete each escape room. Escape room facilitators included the 3 medical students who designed and developed the event. During the event, the facilitators guided the students between rooms once they had “escaped” and reset the puzzles between rotations of groups to allow the escape rooms to be reused. The rooms were self-guided but followed a natural sequence, and facilitators were available for assistance when needed. Participants were advised to knock on the door if they needed help from facilitators with a puzzle. Learners had unlimited opportunities to ask for help, and if they did, they were expected to explain their thought process with the puzzle, then receive guidance regarding the knowledge gap or logical fallacy. All rooms were timed, and prizes were awarded to the winning team with the fastest overall completion time across the 3 rooms. Escape room puzzle materials, setup instructions, and preparation time are provided in Appendices C-E.

### Evaluation Instruments

Participants completed 3 newly developed surveys: a preevent survey assessing knowledge, skills, and attitudes regarding health insurance (Appendix F); an immediate postevent survey evaluating reactions to the escape room modality and interprofessional collaboration (Appendix G); and a 4-month follow-up survey reassessing knowledge, skills, and attitudes (Appendix F). The knowledge assessment (14 multiple-choice questions) included key terms and definitions sourced from the Centers for Medicare and Medicaid Services (CMS) and Healthcare.gov regarding the basics of health insurance terminology and specific inclusion criteria and coverage for Medicare and Medicaid.^15-17^ These questions were chosen based on the lecture material and the most commonly used terminology in patient care settings as assessed by the authors. Knowledge of barriers to patient care was assessed with a mini-survey (3 questions on a Likert scale). The self-efficacy assessment (7 questions on a Likert scale) measured confidence in identifying barriers to patient care and community resources and knowledge of health systems. Our subject matter experts who delivered the lectures reviewed our surveys for content validity to ensure alignment with lecture material.

### Data Collection and Analysis

Each participant was assigned a unique ID code for deidentification. Knowledge scores were calculated by adding the number of correct responses (range 0-14). Self-efficacy was scored on a 4-point Likert scale (range 0-21). Specific scoring criteria are defined in Appendix F. Internal reliability for both scales was confirmed with Cronbach's alpha test > .80. Student knowledge of patient barriers to health care was measured using a 3-question mini-survey (range: 0-3). Paired *t*-tests were performed for the 3 pre- and postevent assessments to evaluate changes in student knowledge, self-efficacy, and patient barriers to care. Given our small sample size, we used the Wilcoxon signed-rank test to perform a sensitivity analysis and evaluate whether conclusions were consistent when using a nonparametric distribution approach. We also calculated effect size using Cohen's *d* for the paired samples.

## Results

### Participant Characteristics

Twenty-nine students from Quinnipiac University participated, including 15 from the Frank H. Netter MD School of Medicine and 14 from other health care professional programs (nursing, occupational therapy, physician assistant, physical therapy, and social work). Most participants were between 20 and 24 years old, female, and primarily of white ethnicity (Table).

**Table.**
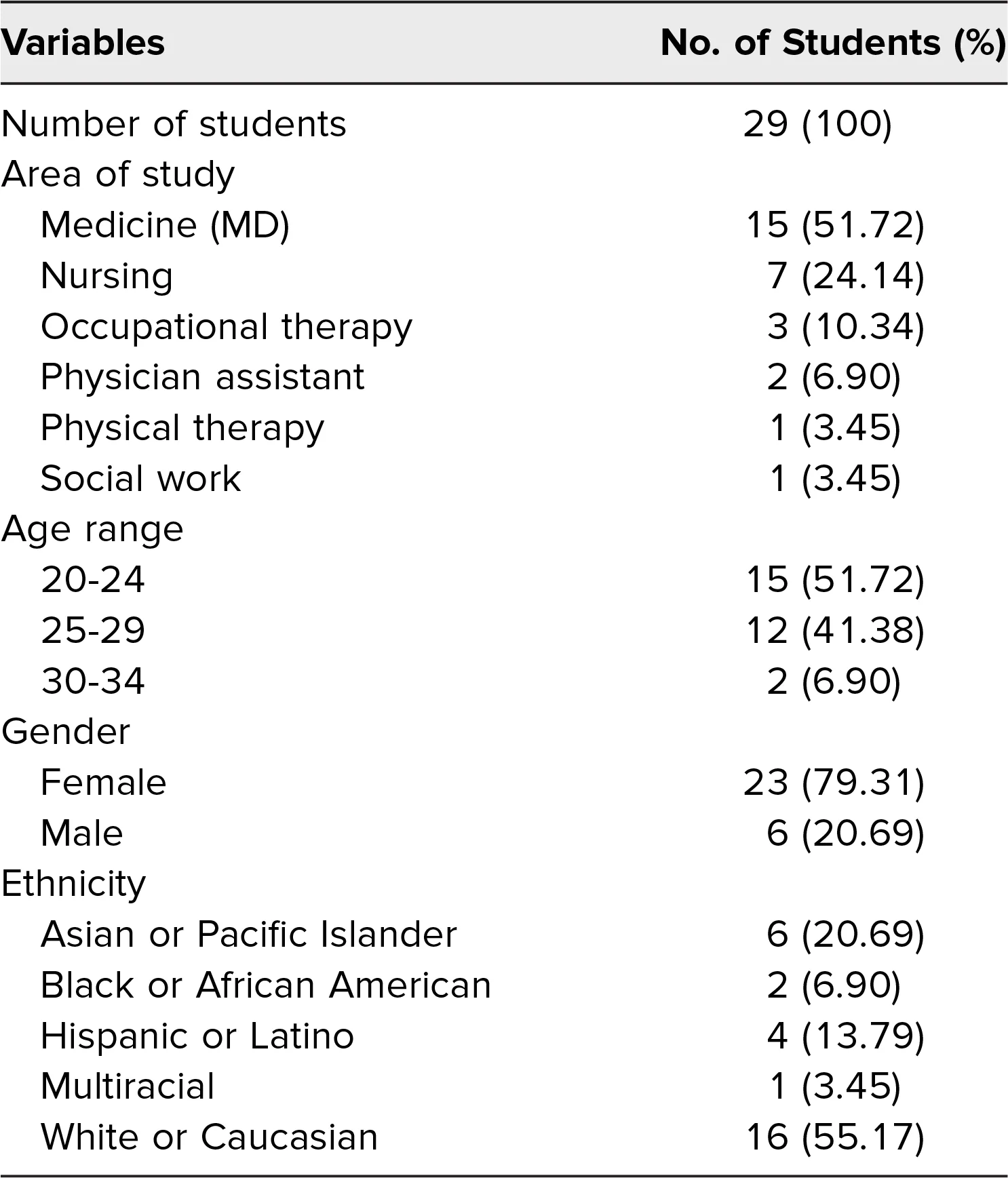
Study Demographics Among Participants (*N* = 29)

### Survey Response Rates

Survey response rates were 100% (*n* = 29) for the preevent survey, 48% (*n* = 14) for the immediate postevent survey, and 59% (*n* = 17) for the 4-month follow-up survey.

### Immediate Reactions (Kirkpatrick Level 1)

Responses to the immediate postevent survey (*n* = 14) demonstrated strong acceptance and feasibility of the escape room modality for teaching health insurance. Participants described the escape room as engaging, enjoyable, interactive, and collaborative while covering crucial material not addressed elsewhere in their curriculum.

All respondents (100%) reported that the event provided them with an opportunity to work with other health profession students, incorporate diverse expertise to address social determinants of health, and engage in teamwork and critical thinking. Most students (93%) reported improved understanding of the roles and capabilities of other health professionals. All groups were able to solve the puzzles and escape the case-based patient room. One student noted the escape room included “creative activities that felt unique and like a great way to review the information.” Key areas of constructive feedback included requests to remove time limits to allow deeper reflection and to provide a reference resource after the event.

### Knowledge Outcomes (Kirkpatrick Level 2)

At 4 months post event, there was no statistically significant improvement in health insurance knowledge compared to baseline. Among medical students (*n* = 10), the mean difference (*MD*) in knowledge was 1.10 (*SD* = 1.97; *P* = .11; Cohen's *d* = 0.56). Among the combined interdisciplinary cohort, including both medical students and other health care students (*n* = 17), the *MD* was 1.18 (*SD* = 2.38; *P* = .06; Cohen's *d* = 0.50). Sensitivity analyses using Wilcoxon signed-rank tests yielded similar conclusions, with no statistically significant differences observed between pre- and postevent scores in either group (*P* = .16; *P* = .09) (Figure 1).

**Figure 1.**
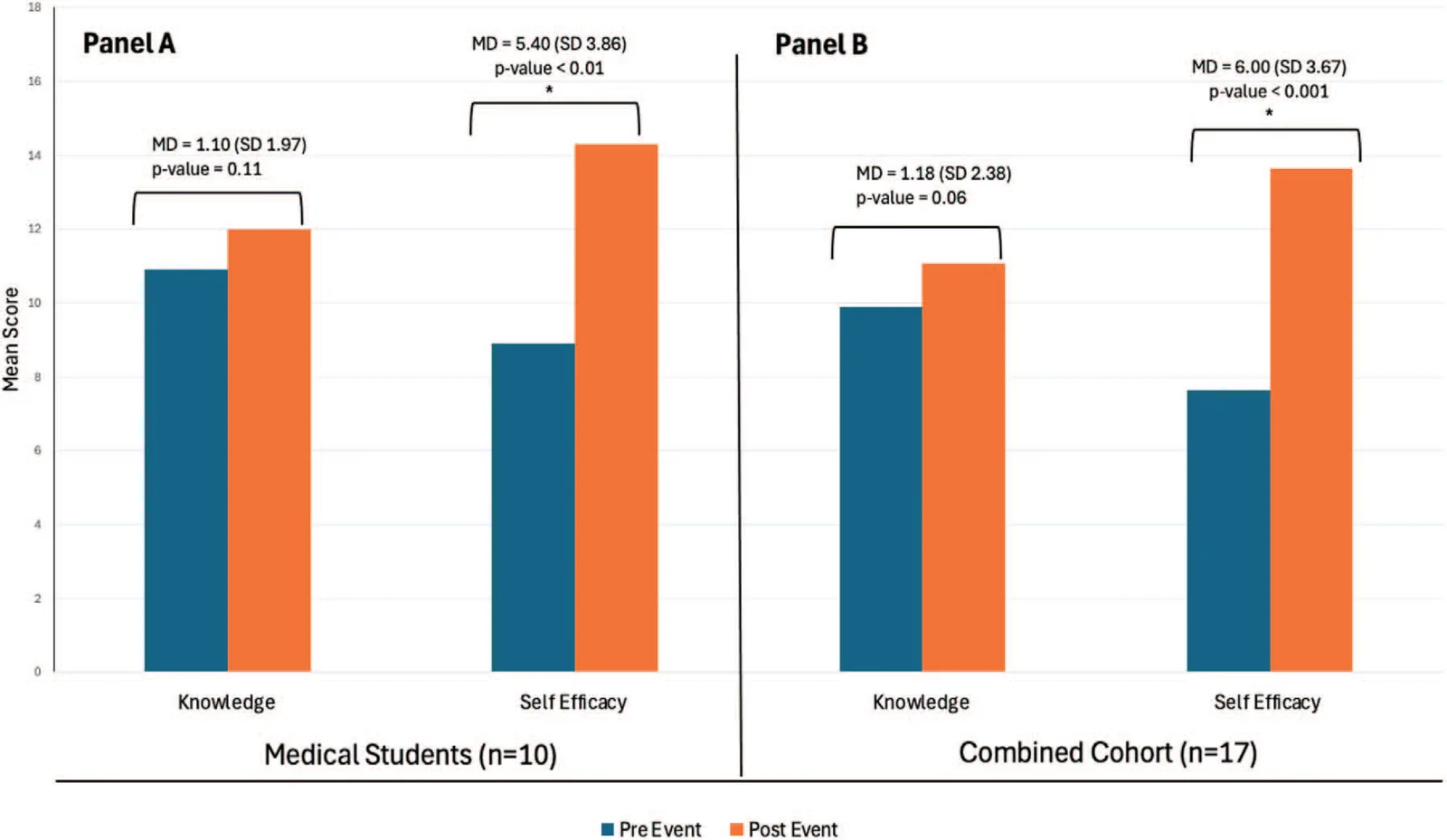
Changes in knowledge and self-efficacy among medical students and the combined interdisciplinary cohort. Mean knowledge scores (range 0-14) and self-efficacy scores (range 0-12) at baseline and 4-month follow-up with reported mean differences (*MD*) and *P* values. Panel A shows medical students (*n* = 10), and Panel B shows the overall combined interdisciplinary cohort (*n* = 17).

### Self-Efficacy Outcomes (Kirkpatrick Level 2)

Both medical students and the combined cohort demonstrated statistically significant increases in self-efficacy scores (*MD* = 5.40, *SD* = 3.86, *P* < .01, Cohen's *d* = 1.40; *MD* = 6.00, *SD* = 3.67, *P* < .001, Cohen's *d* = 1.63). These results remained significant in sensitivity analyses with Wilcoxon signed-rank tests (*P* < .01; *P* < .01) (Figure 1).

### Barriers-to-Care Outcomes (Kirkpatrick Level 2)

Learners’ knowledge of barriers to patient care significantly increased in the domain of patient lack of control over factors that affect their health (*MD* = 0.47; *SD* = 0.874; Cohen's *d* = 0.538; *P* = .04). However, this result did not remain significant in the sensitivity analysis, with a *P*-value of .08 in Wilcoxon signed-rank tests, suggesting the results should be interpreted with caution. Knowledge of the other 2 domains did not differ significantly from baseline (*MD* = 0.24, *SD* = 0.75, Cohen's *d* = 0.31, *P* = .22; *MD* = 0.24, *SD* = 0.66, Cohen's *d* = 0.35, *P* = .16) (Figure 2).

**Figure 2.**
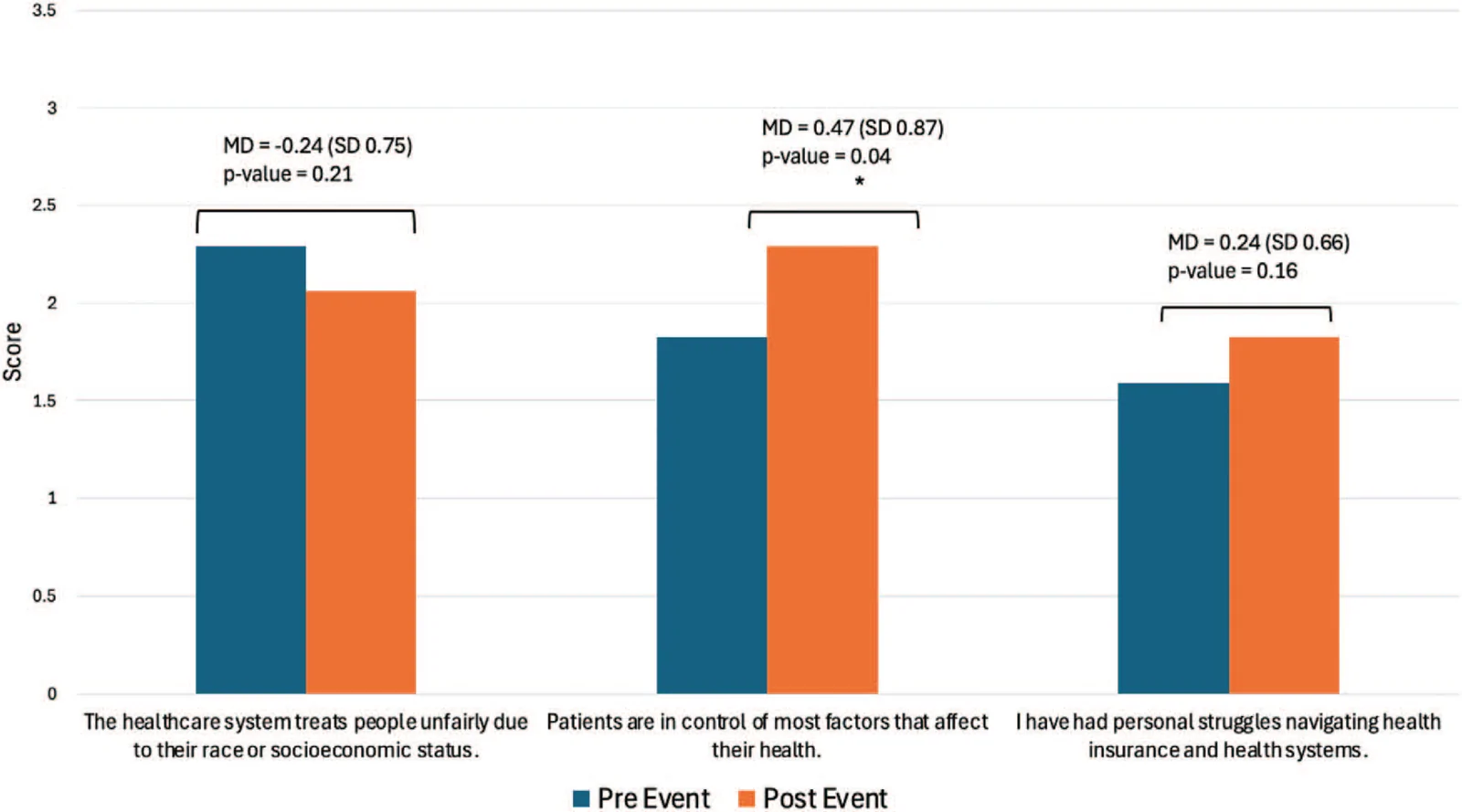
Combined interdisciplinary cohort knowledge of patient barriers. Mean scores on a 3-item barriers-to-care survey (range 0-3) among the overall combined interdisciplinary cohort (*n* = 17) with reported mean differences (*MD*) and *P* values.

## Discussion

Our 2-part interprofessional lecture and escape room educational event is one of few studies exploring the use of escape room-based learning in medical education, and it is the first to utilize this modality for teaching health insurance. The study sample (*n* = 29) was well balanced between medical students (*n* = 15) and other health professional students (*n* = 14). The escape room simulation was associated with significant improvements in learner self-efficacy on health insurance topics across both medical students and the broader cohort. Knowledge outcomes demonstrated small to moderate improvements but did not achieve statistical significance, suggesting knowledge gains may require a larger sample size or additional education to detect. All participants successfully worked in interprofessional teams and addressed barriers to care to complete the puzzles. Overall, our educational event successfully helped learners meet educational objectives 2-4.

A key lesson learned was that self-efficacy improved even without significant knowledge gains, suggesting that confidence building may precede knowledge retention. Self-efficacy may increase when learners feel more capable of applying concepts in practice, even if factual recall does not dramatically increase. This is consistent with the Dunning-Kruger effect described in the medical education literature, in which learning often begins with high self-confidence despite low performance. The model suggests that experience enables learners to recognize their limitations, identify knowledge gaps, and develop competence.^18^ The isolated improvement in self-efficacy in our study may also reflect the limited sensitivity of the knowledge assessment or the limited statistical power due to the small sample size.

Beyond health insurance knowledge, the escape room promoted interprofessional teamwork and problem solving with collaboration and shared understanding of the roles of the health care team to directly address the third and fourth educational objectives. For example, in the Medicaid escape room (Appendix C), learners had to work together to overcome barriers to patient care, including food insecurity, transportation, and lack of insurance, by connecting the patient to appropriate community resources. Learners reported opportunities to collaborate across professions and apply diverse expertise to address social determinants of health, demonstrating the value of interprofessional teamwork in patient care. These findings underscore the value of IPE frameworks in preparing students for team-based patient care.

### Limitations

Limitations include the small sample size, a relatively short follow-up period of 4 months, and a postsurvey response rate of 59%. Because our evaluation relied on self-reported surveys, results may be influenced by social desirability bias, recall bias, and response bias. The small sample size, in particular, may have limited the ability to detect statistically significant changes in knowledge despite observed trends.

### Lessons Learned for Educators

This innovation was developed and implemented by 3 medical students with faculty support. Beyond feasibility, this innovation illustrates how student-driven design can be scaled and adapted across institutions. To replicate this event, we suggest at least 1 facilitator per individual room in the escape room portion. We had 1 facilitator assigned to run 2 rooms simultaneously and felt that additional support would be helpful to maximize efficiency and to minimize the time learners spent between escape rooms.

The escape room format is likely transferable. Educators can substitute local insurance policies, adapt puzzles to different learner levels, and embed the activity within existing interprofessional curricula. By providing detailed appendices with facilitator scripts, puzzle materials, and evaluation instruments, we ensure reproducibility. We primarily utilized the state insurance website in addition to university-based clinicians with real-world experience to frame the lecture portion of our educational event. Replication at other institutions would also require key components: a capable team of facilitators, appropriately knowledgeable lecturers, and sufficient space for the escape rooms. For institutions with standardized patient rooms, this serves as a natural setting for the case-based escape rooms; however, a classroom with tables and cabinets could also be modified to support the puzzles.

Other modifications, suggested by student feedback, include making the escape room untimed and providing a postevent educational resource for reference. The original time constraint was intentionally designed to mimic commercial escape rooms and introduce a sense of competition, thus motivating learners to work efficiently. However, for novice learners, the time-limited structure may have created unnecessary mental processing demands, potentially constraining their ability to fully process and apply new concepts. This pressure may have contributed to the limited knowledge gains observed.

Spaced repetition, particularly when paired with open-ended questions for self-testing, has been shown to be efficacious in knowledge retention compared to traditional studying techniques.^19,20^ Future iterations could incorporate a required postevent learning module with open-ended questions to reinforce knowledge through spaced repetition. If implemented as an optional assignment, variable learner engagement, as seen with our optional preevent work, may limit the depth and consistency of knowledge reinforcement.

### Future Directions and Generalizability

Future iterations of similar work should consider larger organizing teams to expand participation and explore broader implementation, such as integration into required medical education experiences and multi-institutional replication. We found it difficult to recruit busy students for this nonrequired event; it may be feasible in future implementations to make it required for learners as part of their curriculum. A required curricular component could also improve survey responses; ours were suboptimal, despite offering gift cards for survey completion. The ability to scale this innovation across institutions could enhance student preparedness to navigate insurance complexities and work in an interprofessional team. Additionally, managing the escape rooms was difficult with just 3 student organizers; additional institutional involvement, faculty facilitation, and a greater number of organizers could help make the implementation more seamless.

Future versions could include annually updated policy modules that reflect changes in Medicaid, Marketplace, and reproductive-health policies. Educators can use CMS updates and notifications to keep cases current and prepare learners to navigate evolving insurance barriers to care.

Ultimately, this work contributes to the broader educational scholarship of health policy and advocacy training. By embedding health insurance education into required curricula and disseminating reproducible escape room materials, educators can collectively advance learner preparedness to navigate insurance complexities and advocate for patients across diverse practice settings.

## Appendices


Preevent Work Patient Perspectives.docxLecture.pptxMedicaid Patient Case.docxMedicare Patient Case.docxInsurance Terms and Definitions Puzzle.docxPre- and Postevent Assessment.docxImmediate Postevent Evaluation.docx
*All appendices are peer reviewed as integral parts of the Original Publication.*
