# Supplementary material for: Teaching Health Insurance and Patient Barriers Through an Interprofessional Escape Room for Preclinical Medical and Health Professional Students
Source: MedEdPORTAL. 2026 Oct 30;22:11647. doi: 10.15766/mep_2374-8265.11647 (PMC13720544; doi:10.15766/mep_2374-8265.11647)
Supplement: Supplementary file 1 [file mep_2374-8265.11647-s001.zip › A. Preevent Work Patient Perspectives.docx]

**Appendix A Pre-Event Work Patient Perspectives**

**Purpose:** This pre-event work was designed to expose learners to diverse patient perspectives on health insurance barriers and care inequities. Learners were encouraged to complete the readings/videos prior to the lecture and escape room activity to enhance appreciation of patient experiences. Completion was optional to reduce burden, but strongly recommended.

**Materials:**

I. It’s Not Just You: Many Americans Face Insurance Obstacles Over Medical Care and Bills. New York Times.[^1^](https://www.zotero.org/google-docs/?6Q332V)
   This 2023 article from the New York Times presents statistics from KFF showing that a majority of Americans have encountered obstacles with health insurance coverage, proving that it’s not a niche problem in our country. These obstacles include denied claims, affordability, and lack of in-network doctors, and are most likely to significantly affect those who are in greater need of medical care. The consequences of these challenges are highly dangerous to health and difficult to overcome.
II. ‘No one should have to be fighting cancer and insurance at the same time’. CNN.[^2^](https://www.zotero.org/google-docs/?nswRY2)
    This 2024 CNN article, published shortly after the shooting of UnitedHealthcare CEO Brian Thompson, describes the rising tensions and anger in Americans struggling with the health insurance system. The interviewed patients share their stories of denied treatments, huge out-of-pocket costs, and endless phone battles they experienced while concurrently fighting serious illnesses. Many were faced with their care being denied or only offered with a massive price tag, even when their medical team argued that it was necessary. This piece shows the conflicts between providers, insurance companies, and patients that define today's healthcare system.
III. No insurance and a catastrophic illness: My father’s story. San Antonio Report.[^3^](https://www.zotero.org/google-docs/?vAtg35)
     Roseanna Garza recounts how her father, a 60-year-old restaurant manager with no health insurance, avoided medical care until a massive stroke left him paralyzed and dependent on an aid program called CareLink that provides care to locals who lack insurance coverage. The one-income family now juggles his ongoing seizures, repeated hospitalizations and bills, and at-home care, largely provided by an untrained son. This story highlights the heavy financial and emotional toll on families when chronic conditions go untreated and insurance coverage is not an affordable or understandable option. Garza argues that paid sick leave, broader insurance coverage, and systemic solutions are essential to prevent low-income patients from delaying care and incurring far costlier, life-altering crises.
IV. Snapshots from the Kitchen Table: Family Budgets and Health Care. Kaiser Family Foundation.[^4^](https://www.zotero.org/google-docs/?ZlxS7Y)

This 2009 Kaiser Family Foundation documentary profiles several American families who are struggling to make ends meet. It depicts the narrow financial ledge on which millions of low- and middle- income working households stand even in normal economic times, and illustrates the central role that health care costs and coverage play in a household’s economic stability. Some of the families profiled have health insurance, others do not.

V. Personal Stories about the U.S. Health Care System. The Susan G. Komen Advocacy Alliance.[^5^](https://www.zotero.org/google-docs/?DTvDtU)
   This 2009 compilation presents dozens of first-person accounts of challenges with the US healthcare system. These stories of struggling with insurance coverage, accessing timely treatment, and being bankrupted by medical debt show the significant gaps in the system and the human cost of policy failures. They paint a picture of the life-altering uncertainty, stress, and heartbreak that are based on access to healthcare.

**References:**

[1.](https://www.zotero.org/google-docs/?MjO4oP)	[Abelson R. It’s Not Just You: Many People Confront Health Insurance Obstacles on Care and Bills - The New York Times. New York Times. June 15, 2023. https://www.nytimes.com/2023/06/15/health/health-insurance-medical-bills.html](https://www.zotero.org/google-docs/?MjO4oP)

[2.](https://www.zotero.org/google-docs/?MjO4oP)	[Luhby T. ‘No one should have to be fighting cancer and insurance at the same time.’ CNN. December 12, 2024. https://www.cnn.com/2024/12/12/business/us-health-care-insurance-frustrations](https://www.zotero.org/google-docs/?MjO4oP)

[3.](https://www.zotero.org/google-docs/?MjO4oP)	[Garza R. No insurance and a catastrophic illness: My father’s story. San Antonio Report. February 17, 2020. https://sanantonioreport.org/no-insurance-and-a-catastrophic-illness-my-fathers-story/](https://www.zotero.org/google-docs/?MjO4oP)

[4.](https://www.zotero.org/google-docs/?MjO4oP)	[Snapshots from the Kitchen Table: Family Budgets and Health Care. KFF. February 1, 2009. https://www.kff.org/uninsured/snapshots-from-the-kitchen-table-family-budgets-and-health-care/](https://www.zotero.org/google-docs/?MjO4oP)

[5.](https://www.zotero.org/google-docs/?MjO4oP)	[Personal Stories about the U.S. Health Care System. *Susan G Komen Advocacy Alliance*. Published online January 2009. https://www.komen.org/uploadedFiles/Content/GetInvolved/Legislation/Public_Policy/Personal_Health_Care_Stories.pdf](https://www.zotero.org/google-docs/?MjO4oP)
