# Supplementary material for: Teaching Health Insurance and Patient Barriers Through an Interprofessional Escape Room for Preclinical Medical and Health Professional Students
Source: MedEdPORTAL. 2026 Oct 30;22:11647. doi: 10.15766/mep_2374-8265.11647 (PMC13720544; doi:10.15766/mep_2374-8265.11647)
Supplement: Supplementary file 1 [file mep_2374-8265.11647-s001.zip › C. Medicaid Patient Case.docx]

**Appendix C: Medicaid Patient Case**

**Facilitator’s Guide**

**Purpose:** This escape room case was designed to simulate a realistic patient scenario requiring Medicaid eligibility knowledge, social services navigation, and genetic counseling referral. Learners apply insurance concepts in a collaborative, problem-solving environment.

**Supplies Needed:**

- Printed materials (see below), cut up according to letter indicators
  - *Note that you will need one copy of the crossword for each group as they will be filling it out*
- 16 magnets
- Magnetic whiteboard
- Tape
- Pen or pencil
- Marker
- Custom insurance card jigsaw puzzle (there are multiple websites that allow you to upload a photo (B) and will send you the puzzle, we suggest one with under 30 pieces)
- 2 hollow books - one labeled “SOCIAL WORK” and one labeled “GENETIC TESTING”
- Several other books with names related to healthcare to serve as distractors
- Small kitchen scale
- Model bus
- Can of soup
- Model brain
- 4 digit bike lock (code set to 0553)
- 3 digit lock box (code set to 210)
- Sticky notes

*(If a whiteboard is not available, this can also be done with the cards on a table.*

*If an entire room is not available, we recommend setting up the puzzles in order across a table.)*

**Set-Up:**

*Estimated time required: 15 minutes for initial set-up; 20 minutes maximum for the students to “escape” the room; 5 minutes to reset the room by rescrambling the puzzle, resetting the locks, and rescrambling the cards; 5 minutes to clean up*

1. Print all the materials below the line.
2. Fasten the student doorway instructions to the outside of the simulated patient room.^A^
3. Write with a marker on the top right corner of the insurance card jigsaw puzzle “What health professional would you call for help? Find them on the shelf.”
4. Scramble the jigsaw puzzle and place it on the exam table. ^B^
5. Leave a sticky note on the exam table with the jigsaw puzzle that says “START HERE!”
6. Print out the social work note and place it in a hollowed out book with a spine labeled “SOCIAL WORK.” Leave the book in a pile with a collection of healthcare books as distractors. ^C^
   a. *Note: Replace with your state-specific programs.*
7. Scatter model bus, soup can, and model brain throughout the room.
8. Leave the small kitchen scale on the counter.
9. Place notes in a cabinet prior to locking: success note for completing social programs problem, note indicating desire for genetic counseling, crossword puzzle with clues. ^D, E, F^
   a. *Note: Replace with your state-specific programs.*
10. Lock up this cabinet with the 4 digit bike lock. Set the code to the corresponding weight of the model bus + soup can + model brain on the kitchen scale (i.e. 553 g = code 0553).
11. Tape the label that says “CHART” on a different cabinet. ^G^
12. Print the patient chart.^H^
13. Highlight the date of DMD diagnosis on the chart (i.e. 2/10). Write “You know DMD is genetic. How could you learn more about your patient's risk?” on a sticky note and place it on the patient chart. Place the patient chart with the sticky note on it in the “CHART” labeled cabinet.
14. Print out the HUSKY (Medicaid) policy document and genetic counselor referral note and place them in the second hollowed out book in the stack labelled “GENETIC TESTING.” Highlight statement number 1 under Genetic Testing on the Medicaid policy document. ^I, J^
    a. *Note: Replace with your state-specific programs.*
15. Write “Your patient wants to learn more about DMD. Find a visual to teach them more about it” on a sticky note and place that in the hollowed out “GENETIC TESTING” book.
16. Tape the DMD visual with the arrow pointing down on the wall above the lock box.^K^
17. Place the 3 digit lock box below the DMD visual. Set the code to the diagnosis date found in the patient chart (i.e. diagnosed 2/10 = code 210).
18. Inside the lockbox, place the HUSKY approval note and the success note prompting the students to move on to the matching game.^L,M^
    a. *Note: Replace with your state-specific programs.*
19. Print and cut the insurance terms and definitions.^N^
    a. *Note: Replace with your state-specific programs.*
20. Write a facilitator’s phone number behind the definition cards (right hand column) in the order they appear below, with one letter per definition card. Write a note on the whiteboard that says the students may only move the definitions, but must leave the terms in place. You can tape them down instead of magnets if desired to ensure this.
21. Arrange the insurance definitions horizontally on the whiteboard below the terms, scrambled. When the students place them in the correct matching order, they should arrive at the correct phone number to call and “escape the room.”
22. Read Facilitator Script. Allow students to read doorway instructions, and enter the room. Set a timer for 20 minutes.
23. Once students escape the room, guide them to a designated break room until it is time to start the next escape room.

**Facilitator Script:**

“Students, welcome to patient case 1. When you enter the patient room, you will have a puzzle to solve with your team members on the exam table. Items that are a part of the escape room puzzle will be easily manipulated; please do not interfere with the integrity of the exam room. If there are any questions during the activity, please open your door and we will be here to assist you. Are there any questions before we begin?”

(Pause for questions.)

“You may begin.”

**Printed Materials:**

---------------------------------------------------------------------------------

(A)

Escape Room Rules:

CASE 1: Medicaid

Your “patient” recently lost her job, is low-income, and is pregnant for the first time. Use your new knowledge to work through the escape room and help her navigate insurance and social programs. **Please start with the indicated puzzle**. Please do not damage the room (clues should be obvious). Exit the same way you came in. If you get stuck, we are just outside to help. Thank you! Work together and have fun!

—-------------------------------------------------------------------------------------------

(B)

Figure B1. Author-created simulated insurance card made using Microsoft Word.^1^

—-------------------------------------------------------------------------------------------

(C)

Great job! With your assistance, the patient was referred to social work and successfully applied for Medicaid, regaining her health insurance. However, she remains pregnant and unemployed, facing daily challenges with **transportation, mental health, and food insecurity**. Luckily, Connecticut’s state programs such as SNAP provide her with additional resources to help her meet her needs. Objects that symbolize these **3** state programs can make a **HEAVY** impact. *Hint: How do you measure something heavy?*

*Note: Replace with your state-specific programs.*

—-------------------------------------------------------------------------------------------

(D)

Congratulations! Thanks to the support of social workers, you successfully helped your patient secure transportation assistance for her appointments, meal assistance to alleviate food cost concerns, and mental health resources to manage the stress of her situation.

—-------------------------------------------------------------------------------------------

(E)

Your patient now expresses a desire for prenatal genetic testing. She suspects her child may be at risk for genetic conditions, but you need support to convince the insurance representatives to cover her treatment. Consider reaching out to **other healthcare professionals** who have worked with this patient; they might be able to help.

—-------------------------------------------------------------------------------------------

(F)

ACROSS

1. You might be in this state often

3. A health provider (doctor, PA, NP, etc.) who serves as a patient’s main point of contact for non-emergency healthcare

5. CT’s public health coverage program encompassing Medicaid and CHIP

6. A health provider who examines, diagnoses, and treats patients under the supervision of a physician (requires 2-3 years of advanced schooling)

7. A health provider who assists patients in childbirth

8. A health provider who cares directly for patients in all settings (types include RN, APRN, and NP)

9. A health provider who counsels patients on diet and nutrition

11. A health provider who is trained in psychology and mental health

DOWN

2. A physician who specializes in pregnancy, childbirth, and the female reproductive system

4. A genetic _____ helps patients understand their risks from inherited conditions

6. A review process done by a physician in which they discuss the need for a patient’s treatment with an insurance company to convince them to provide authorization

10. A health provider who evaluates and treats people who have conditions or injuries that limit their ability to move and do physical activities

Figure F1. Author-created crossword puzzle and answer key made using Microsoft Word.^2^

*Note: Replace with your state-specific programs.*

—-------------------------------------------------------------------------------------------

(G)

CHART

—-------------------------------------------------------------------------------------------

(H)

Figure H1. Author-created using Microsoft Word.^3^

—-------------------------------------------------------------------------------------------

(I)

Figure I1. Author created image made in Microsoft Word.^4^

Reference material^5^:

Policies, procedures, & guidelines. HUSKY Health Program | HUSKY Health Providers | Policies, Procedures, and

Guidelines. Accessed March 5, 2026. https://www.huskyhealthct.org/providers/policies_procedures.html.

*Note: Replace with your state-specific program.*

—-------------------------------------------------------------------------------------------

(J)

Figure J1. Author-created patient referral using Microsoft Word.^6^

—-------------------------------------------------------------------------------------------

(K)

Figure K1. Author-created using Microsoft Word.^7^

Reference material^8^:

Venugopal V, Pavlakis S. Duchenne Muscular Dystrophy. [Updated 2023 Jul 10]. In: StatPearls [Internet].

Treasure Island (FL): StatPearls Publishing; 2025 Jan-. Available from: https://www.ncbi.nlm.nih.gov/books/NBK482346/

Figure K2. Author-created using Microsoft Word. ^9^

—-------------------------------------------------------------------------------------------

(L)

04/03/2025

Dear Francesa Netterson,

Since you have established family history of genetic disease, have met with genetic counseling, and have the test ordered by a certified physician, the HUSKYHealth Team has approved your request for prenatal genetic testing.

*Note: Replace with your state-specific programs.*

—-------------------------------------------------------------------------------------------

(M)

Congratulations! The patient now qualifies for prenatal genetic testing covered by HUSKY insurance, due to her family history of Duchenne’s muscular dystrophy. However, she is still confused about what CT social programs are available to her and her future child. With your new knowledge, you feel confident explaining these programs to her. Match the correct terms with their definitions on the whiteboard. If they are placed correctly, the patient's phone number will be revealed. Call this number to reach her.

*Note: Replace with your state-specific programs.*

—-------------------------------------------------------------------------------------------

(N)

| **1**    **HUSKY A** | Children, their parents, and pregnant women |
| --- | --- |
| **2**    **HUSKY B** | Uninsured children under age 19 in higher-income households |
| **3**    **HUSKY C** | 65 years of age or older, and/or who are blind or disabled |
| **4**    **HUSKY D** | Aged 19 up to 65th birthday without dependent children; who do not receive Medicare; and who are not pregnant |
| **5**    **Supplemental Nutrition Assistance Program**  **(SNAP)** | Monthly money on a debit-like EBT card to buy food |
| **6**    **Connecticut Energy Assistance Program (CEAP)** | Benefits towards your heating bill |
| **7**    **Connecticut Home Care Program for Elders (CHCPE)** | Encompasses programs like Adult Day Health Services, Companion Services, Home Delivered Meals, Homemaker Services |
| **8**    **Child Care Assistance** | Establishing parentage, enforcing child support orders, locating noncustodial parents |

*Note: Replace with your state-specific programs.*

**References:**

1. Devkota, K. (2026). Simulated insurance card for escape room activity. Created using Microsoft Word.
2. Spencer, L. (2026). Crossword puzzle for escape room activity. Created using Microsoft Word.
3. Spencer, L. (2026). Simulated patient chart. Created using Microsoft Word.
4. Spencer, L. (2026). Document providing information about Husky coverage for genetic testing. Created using Microsoft Word.
5. Policies, procedures, & guidelines. HUSKY Health Program | HUSKY Health Providers | Policies, Procedures, and Guidelines. Accessed March 5, 2026. https://www.huskyhealthct.org/providers/policies_procedures.html.
6. Spencer, L. (2026). Simulated patient referral. Created using Microsoft Word.
7. Spencer, L. (2026). Infographic. Created using Microsoft Word.
8. Venugopal V, Pavlakis S. Duchenne Muscular Dystrophy. [Updated 2023 Jul 10]. In: StatPearls [Internet]. Treasure Island (FL): StatPearls Publishing; 2025 Jan-. Available from: <https://www.ncbi.nlm.nih.gov/books/NBK482346/>
9. Spencer, L. (2026). Arrow. Created using Microsoft Word.
