# Supplementary material for: Teaching Health Insurance and Patient Barriers Through an Interprofessional Escape Room for Preclinical Medical and Health Professional Students
Source: MedEdPORTAL. 2026 Oct 30;22:11647. doi: 10.15766/mep_2374-8265.11647 (PMC13720544; doi:10.15766/mep_2374-8265.11647)
Supplement: Supplementary file 1 [file mep_2374-8265.11647-s001.zip › D. Medicare Patient Case.docx]

**Appendix D: Medicare Patient Case**

**Facilitator’s Guide**

**Purpose:** This escape room case was designed to simulate a realistic patient scenario requiring Medicare coverage knowledge, surgical eligibility determination, and post-operative care planning. Learners apply insurance concepts in a collaborative, problem-solving environment while reinforcing interdisciplinary teamwork.

**Supplies Needed:**

- Printed materials (see below), cut up according to letter indicators
- 12 magnets
- Magnetic whiteboard
- Tape
- Sticky note that says “START HERE! Begin by finding your patient’s PCP.”
- Business cards
- Prescription pill bottle (attach printed label)
- Poster/photograph of a bridge (write the number 738 on the corner)
- Lock box (set code to 738)
- Operation game and batteries
- Book about Deep Vein Thrombosis (you can buy hollow books or modify existing books)

*(If a whiteboard is not available, this can also be done with the cards on a table.*

*If an entire room is not available, we recommend setting up the puzzles in order across a table.)*

**Set-Up:**

*Estimated time required: 15 minutes for initial set-up; 20 minutes maximum for the students to “escape” the room; 5 minutes to reset the room by resetting the locks, putting the notes back in the Operation game, and rescrambling the cards; 5 minutes to clean up*

1. Print all materials below the line.
2. Fasten the student doorway instructions to the outside of the simulated patient room.^A^
3. Spread the business cards on a counter. Place the sticky note next to them. ^B^
4. Put the labeled prescription pill bottle nearby. Students should be able to read the prescription label on the pill bottle and put that information together with the business cards to determine the name of the PCP. ^C^
5. Tape the labels that say “Patient Files” on different cabinets, one on each cabinet. ^D^
6. Inside the cabinet that says ND Patient Files, place the printed paragraph about Physician Assistants.^E^
7. Inside the cabinet that says BF Patient Files, place the printed paragraph about Nurse Practitioners. ^F^
8. Inside the cabinet that says JS Patient Files, place the Visit Summary for the patient. ^G^
9. Tape the poster of the bridge up on a wall. Make sure the number 738 is written in small text on it.
10. Place the lockbox underneath the bridge poster. Make sure it unlocks if the code 738 is entered.
11. Place the hip X-rays inside the lockbox. ^H^
12. Place the Medicare Local Coverage Determination for Total Hip Arthroplasty inside the lockbox. ^I^
    a. *Note: Replace content with your state-specific requirements as applicable.*
13. Place the Operation game on the patient’s bed (to create an “operating table”).
14. Crumple up the congratulations note and put it into the hole near the hip in the Operation game. Crumple up the other notes and put them in the ribs, stomach, and heart holes so that it is not too easy to determine which one to grab. ^J^
15. Underneath the Operation game or underneath the table, tape the post-surgery physical therapy assessment and recommended exercises. ^K^
16. Rip out part of the recommended exercises (the ankle pumps, quad holds, heel slides, and bridges) so that only one of them is left attached to the normal paper. Rip apart and crumple the three you ripped out then scatter them around the room. We printed them on pink paper to make it obvious they were all part of the same document, but you can use other creative options to signify this.
17. When students put together the exercises, they should notice that the highlighted letters spell “NO DVT.” Place a book about Deep Vein Thrombosis on a shelf in the room.
18. Inside the book, place the congratulations note and the note about insurance bills. ^L^
19. Cut out the insurance terms and definitions and arrange them horizontally in order on the whiteboard with magnets. ^M^
20. Write a facilitator’s phone number behind the definition cards (right hand column) in the order they appear below, with one letter per definition card. Write a note on the whiteboard that says the students may only move the definitions, but must leave the terms in place. You can tape them down instead of magnets if desired to ensure this.
21. Arrange the insurance definitions horizontally on the whiteboard below the terms, scrambled. When the students place them in the correct matching order, they should arrive at the correct phone number to call and “escape the room.”
22. Read Facilitator Script. Allow students to read doorway instructions, and enter the room. Set a timer for 20 minutes.
23. Once students escape the room, guide them to a designated break room until it is time to start the next escape room.

**Facilitator Script:**

“Students, welcome to patient case 2. When you enter the patient room, you will have a puzzle to solve with your team members on the counter. Items that are a part of the escape room puzzle will be easily manipulated; please do not interfere with the integrity of the exam room. If there are any questions during the activity, please open your door and we will be here to assist you. Are there any questions before we begin?”

(Pause for questions.)

“You may begin.”

**Printed Materials:**

------------------------------------------------------------------------------------

(A)

Escape Room Rules:

CASE 2: Medicare

Your “patient” is elderly and has Medicare insurance. She has a history of hip arthritis and associated pain. Use your new knowledge to help her navigate the medical system. **Please start with the indicated puzzle**. Please do not damage the room (clues should be obvious). Exit the same way you came in. If you get stuck, we are just outside to help. Thank you! Work together and have fun!

—-------------------------------------------------------------------------------------------

(B)

Figure B1. Author-created simulated MD business card made using Microsoft Word.^1^

Figure B2. Author-created simulated NP business card made using Microsoft Word.^2^

Figure B3. Author-created simulated PA-C business card made using Microsoft Word.^3^

(C)

| ***Quinnipiac Pharmacy***  1234 Main Street  North Haven, CT 06473  **INTERSON, CARA**  Generic Medication  Take one tablet by  mouth once daily.  **DR. JUSTIN SURANCE** | ***Quinnipiac Pharmacy***  1234 Main Street  North Haven, CT 06473  **INTERSON, CARA**  Generic Medication  Take one tablet by  mouth once daily.  **DR. JUSTIN SURANCE** |
| --- | --- |

(D)

JS Patient Files

ND Patient Files

BEF Patient Files

(E)

Physician Assistants (PAs) are highly skilled healthcare providers who can serve as primary care providers (PCPs). They are trained to diagnose and treat illnesses, manage chronic conditions, and emphasize preventative care. They conduct physician exams, order and interpret tests, counsel on preventative health care, assist in surgeries, and write prescriptions. PAs are integral to providing comprehensive care and improving patient access, especially in underserved areas. However, in this particular case, the patient is seeing a different PCP. Try again!

(F)

Nurse Practitioners (NPs) are advanced practice registered nurses who can serve as primary care providers (PCPs). They diagnose, treat, and manage acute and chronic diseases, emphasizing health promotion and disease prevention. NPs can practice independently in many states, providing a wide range of services, including prescribing medications and coordinating care. Their holistic approach often includes patient education and counseling. However, in this particular case, the patient is seeing a different PCP. Try again!

(G)

**Visit Summary from Last Appointment for Cara Interson**

Physical Exam findings significant for swelling, pain on palpation, limited range of motion for hip flexion/extension, abduction/adduction, and internal/external rotation.

Patient has been following conservative treatment consisting of biweekly physical therapy including **BRIDGES** and Ibuprofen as needed. Conservative treatment unsuccessful at this point.

Figure G1. Pharmacy symbol from Pixabay, used under the Pixabay License.^4^

(H)

**Cara Interson X-RAYS:**

Figure H1. Hip radiograph. Image by Dr. Johannes Sobotta, used under CC BY 3.0.^5^

Figure H2. Hip illustration. Image by OpenStax, used under CC BY 4.0.^6^

(I)

**CMS**.gov Centers for Medicare & Medicaid Services ^7^

| **MCD**  Medicare Coverage Database ^7^ | Local Coverage Determination (LCD)  **LCD - Total Hip Arthroplasty (L34163)**.^8^ |
| --- | --- |

***Will your patient’s insurance cover her surgery?***

***If so, find where it would be done.***

**Total Hip Arthroplasty (THA)**.^8^

Total hip replacement surgery will be considered medically necessary in the following circumstances:

Advanced joint disease demonstrated by:

- Radiographic supported evidence or when conventional radiography is not adequate (subchondral cysts, subchondral sclerosis, periarticular osteophytes, joint subluxation, severe joint space narrowing, avascular necrosis); AND
- Pain that cannot be adequately controlled despite optimal conservative treatment or functional disability from injury due to trauma or arthritis of the joint; AND
- If appropriate, history of unsuccessful conservative therapy (non-surgical medical management) that is clearly addressed in the pre-procedure medical record. Non-surgical medical management is usually but not always implemented prior to scheduling total joint surgery. Non-surgical treatment as clinically appropriate for the patient’s current episode of care typically includes one or more of the following:
  - anti-inflammatory medications or analgesics, or
  - flexibility and muscle strengthening exercises, or
  - supervised physical therapy [Activities of daily living (ADLs) diminished despite completing a plan of care]^8^

*Note: Although CMS.gov is a federal agency, each state program may have different requirements/regulations. Replace with your state-specific content as applicable.*

(J)

Congratulations! Your patient met the Medicare eligibility criteria for total hip arthroplasty. The surgery was successful and free of complications. Look below the operating equipment to see what’s next for your patient.

Not quite, your patient does need surgery, but there is nothing wrong with her ribs.

Not quite, surgery can make people nervous, but your patient does not have butterflies in her stomach.

Not quite, your patient may be old enough to qualify for Medicare, but she does not have a broken heart.

(K)

PhysicalTherapy

Patient Name: Cara Interson

MRN: 98765432

Date: April 3, 2025

Diagnosis: Post Total Hip Arthroplasty (Right Hip)

Subjective: Patient reports mild discomfort in the right hip but states that pain is manageable with prescription medication. She is motivated to follow the rehabilitation plan and is eager to regain mobility.

Objective:

- Vital Signs: Stable
- Incision Site: clean, dry, and intact with no signs of infection
- Range of Motion (ROM):
  - Hip flexion: 0-90 degrees
  - Hip abduction: 0-30 degrees
  - Hip extension: 0-10 degrees
- Strength:
  - Quadriceps: 4/5
  - Hamstrings: ⅘
- Gait: Ambulating with a walker, weight-bearing as tolerated (WBAT).

Assessment: Patient is progressing well post-surgery. No complications observed. Emphasis on thrombosis prevention and gradual increase in activity levels.

Plan:

1. Post-Operative Instructions:

- Continue using walker for ambulation
- Avoid hip flexion beyond 90 degrees, adduction, and internal rotation to prevent dislocation.
- Follow weight-bearing as tolerated (WBAT) guidelines.

2. Exercises:

a**N**kle pumps - 10-15 every h**O**ur

qua**D** holds - 5 second hold, 10 reps, 3 times a day

heel slides - 3 sets of 10 e**V**ery day

bridges - **T**hree sets of 10 every day

Next Appointment: Follow-up physical therapy session scheduled for April 7, 2025.

Signature: Phil Payne, DPT

***A major concern for patients post-hip-replacement is ______. Find all of the exercises to determine what it is, then look for a source of more information about the topic.***

Figure K1. Silhouette icon. Image from Pixabay, used under the Pixabay License.^9^

(L)

Congratulations! Your patient has successfully completed post-op physical therapy. They have returned to their normal daily activities and are no longer experiencing pain.

Your patient is pleased with the results of her surgery and physical therapy. However, she is now receiving insurance bills in the mail and is confused by the various costs and complex terminology. With your new knowledge, you feel confident explaining these terms to her. Match the correct terms with their definitions on the whiteboard. If they are placed correctly, the patient's phone number will be revealed. Call this number to reach her.

(M)

| **1**    **Premium** | Monthly/quarterly or annual payment to maintain coverage |
| --- | --- |
| **2**    **Deductible** | Amount a policyholder must pay out of pocket for covered healthcare services before insurance starts to contribute |
| **3**    **Copay** | Fixed payment for visits |
| **4**    **Out of Pocket Maximum** | Includes deductibles, co-pays, and co-insurance. Must reach this number before 100% coverage |
| **5**    **In-Network** | Doctors, hospitals that have a contract with your insurance company to provide services at a lower, negotiated rate |
| **6**    **Dependent** | Spouses, children, other eligible family members included on your insurance plan |

**References:**

1. Schlak, R. (2025). Simulated MD business card for escape room activity. Created using Microsoft Word.
2. Schlak, R. (2025). Simulated PA-C business card for escape room activity. Created using Microsoft Word.
3. Schlak, R. (2025). Simulated NP business card for escape room activity. Created using Microsoft Word.
4. Clker-Free-Vector-Images. (n.d.). Pharmacy symbol [Image]. Pixabay. <https://pixabay.com/images/id-297390/>. Pixabay License.
5. Sobotta, J. (2008). LeggCalvePerthes2 [X-ray]. Wikimedia Commons. <https://commons.wikimedia.org/wiki/File:LeggCalvePerthes2.jpg>. Licensed under CC BY 3.0.
6. OpenStax. (2016). 0910 Osteoarthritis of the hip - x-ray showing osteoarthritis of the hip [Image]. Wikimedia Commons. <https://commons.wikimedia.org/wiki/File:0910_Oateoarthritis_Hip.jpg>. Licensed under CC BY 4.0.
7. Centers for Medicare & Medicaid Services: CMS.gov. <https://www.cms.gov>.
8. Centers for Medicare & Medicaid Services. Local Coverage Determination (LCD): Hip Arthroplasty (L34163). Medicare Coverage Database. CMS.gov. <https://www.cms.gov/medicare-coverage-database/view/lcd.aspx?LCDId=34163&ver=33>.
9. Clker-Free-Vector-Images. (n.d.). Man silhouette icon [Image]. Pixabay. <https://pixabay.com/vectors/man-running-silhouette-black-40739/>. Pixabay License.
