# Supplementary material for: Teaching Health Insurance and Patient Barriers Through an Interprofessional Escape Room for Preclinical Medical and Health Professional Students
Source: MedEdPORTAL. 2026 Oct 30;22:11647. doi: 10.15766/mep_2374-8265.11647 (PMC13720544; doi:10.15766/mep_2374-8265.11647)
Supplement: Supplementary file 1 [file mep_2374-8265.11647-s001.zip › E. Insurance Terms and Definitions Puzzle.docx]

**Appendix E: Insurance Terms and Definitions Puzzle**

**Facilitator’s Guide**

**Purpose:** This puzzle station was designed to reinforce learner knowledge of key insurance terminology and definitions. By matching terms with their correct definitions, learners consolidate foundational knowledge and “unlock” a hidden message that guides them to a break station.

**Supplies Needed:**

- Printed materials (see below)
- 20 magnets
- Whiteboard
- Marker

*(If a whiteboard is not available, this can also be done with the cards on a table.)*

**Set-Up:**

*Estimated time required: 10 minutes for initial set-up; 20 minutes maximum for the students to “escape” the room; 1 minute to reset the room by shuffling the definition cards; 3 minutes to clean up*

1. Print “Mystery Station” (student instructions) and fasten to the outside of the simulated patient room.
2. Print and cut the insurance terms and definitions.
3. Write the message “BREAK ROOM!” behind the definition cards (right hand column) in the order they appear below, with one letter per definition card.
4. Arrange the insurance terms horizontally in order on the whiteboard with magnets (these must stay in their current order so that the message will be correctly revealed).
5. Arrange the insurance definitions horizontally on the whiteboard below the terms, in no particular order.
6. Write instructions on the whiteboard **“Match the definitions to the correct terms moving the definitions only. Do not move the cards that have the terms on them. Once you have matched all definitions with their terms, flip over the definition cards to reveal a message!”**
7. Read Facilitator Script. Allow students to read doorway instructions, and enter the room. Set a timer for 20 minutes.
8. Once students correctly match the cards to reveal the “BREAK ROOM!” message on their backs,escape the room, guide them to a designated break room until it is time to start the next escape room.

**Facilitator Script:**

“Students, welcome to the mystery station. When you enter the patient room, you will have a puzzle to solve with your team members on the whiteboard. If there are any questions during the activity, please open your door and we will be here to assist you. Are there any questions before we begin?”

(Pause for questions.)

“You may begin.”

**Printed Materials:**

-----------------------------------------------------------------------------------

MYSTERY STATION

Spend this time applying your new knowledge to unlock a secret message.

-----------------------------------------------------------------------------------

| **1**    **CHIP** | Covers children in low-income families who do not quality for Medicaid |
| --- | --- |
| **2**  **Health Maintenance Organization (HMO)** | Requires primary care physician referral, lower premiums and out of pocket costs, requires use of in-network providers |
| **3**  **Preferred Provider Organization (PPO)** | No referral needed for specialists, higher premiums and out-of-pocket costs, but more flexibility in choosing providers |
| **4**    **Point of Service (POS)** | Hybrid HMO and PPO, lower cost in-network and higher cost out of network |
| **5**    **Medicare** | Federal program for seniors (65+) and certain disabled individuals |
| **6**    **Medicare Part A** | Hospital insurance |
| **7**    **Medicare Part B** | Medical insurance |
| **8**  **Medicare Part C (Advantage)** | Private plans offering additional benefits |
| **9**    **Medicare Part D** | Prescription drug coverage |
| **10**    **Medicaid** | Joint federal-state program for low-income individuals and families |
