# Supplementary material for: Teaching Health Insurance and Patient Barriers Through an Interprofessional Escape Room for Preclinical Medical and Health Professional Students
Source: MedEdPORTAL. 2026 Oct 30;22:11647. doi: 10.15766/mep_2374-8265.11647 (PMC13720544; doi:10.15766/mep_2374-8265.11647)
Supplement: Supplementary file 1 [file mep_2374-8265.11647-s001.zip › F. Pre- and Postevent Assessment.docx]

**Appendix F: Pre- and Post-Event Knowledge and Self-Efficacy Assessment**

**Purpose:** This survey instrument was designed to evaluate learner knowledge, self-efficacy, and attitudes regarding health insurance and barriers to care. It was administered pre-event and four months post-event to assess retention.

**Scoring:**

- Knowledge Assessment: Assign 1 point for each correct answer (total score: 0-14). For multiple-response questions (#4, 6, 7, 8, and 11), participants must select all correct options to receive credit.
- Self-efficacy Assessment: Score responses on a 0–3 scale (0 = Strongly Disagree; 3 = Strongly Agree). Total score (0-21).
- Barriers to Patient Care Assessment: Score responses on a 0–3 scale (0 = Strongly Disagree; 3 = Strongly Agree). Reverse code Question 2 (0 = Strongly Agree; 3 = Strongly Disagree).

**Informed Consent:**

1. I have read the consent form and I understand that it is up to me whether or not I participate. I know enough about the purpose, methods, risks and benefits of the research study to decide that I want to take part in it. I understand that I am not waiving any of my legal rights by signing this informed consent document.
   a. Yes
   b. No

**Demographics:**

1. What is your area of study?
   a. Medicine (MD)
   b. Nursing
   c. Occupational Therapy
   d. Physical Therapy
   e. Physician Assistant
   f. Social Work
   g. Other (please specify): _____
2. What is your age range?
   a. 20-24
   b. 25-29
   c. 30-34
   d. 35–39
   e. 40+
3. What gender do you most identify with?
   a. Cisgender Female
   b. Cisgender Male
   c. Non-binary
   d. Transgender Female
   e. Transgender Male
   f. Other (please specify): _____
   g. Prefer not to say
4. What would best describe you?
   a. Asian or Pacific Islander
   b. Black or African American
   c. Hispanic or Latino
   d. Native American or Alaska Native
   e. White or Caucasian
   f. Other (please specify): _____
5. Have you personally had difficulties getting coverage for a medication or treatment you needed as a patient?
   a. Yes, I had difficulties with health insurance as a patient
   b. No, I have not had difficulties with health insurance as a patient
6. Have you received any prior formal education about health insurance?
   a. Yes, I have taken a class or received another form of formal education about health insurance
   b. No, I have not had prior formal education about health insurance

Knowledge Assessment: (multiple choice, select all that apply). Correct answers in **bold**.

1. What is a premium?
   a. **The amount that must be paid for your health insurance plan monthly, quarterly or yearly by you and/or your employer.**
   b. The cost of a prescription drug.
   c. The amount paid for medical services at the time of a procedure or treatment.
   d. The amount a healthcare provider charges for a visit to their office for an appointment.
2. What is a deductible?
   a. The initial cost of your healthcare premiums each month.
   b. The total amount your insurance company will pay for your medical expenses in a year.
   c. The percentage of medical costs covered by your insurance after a certain limit is reached.
   d. **The amount you owe for health care services your health insurance or plan covers before your health insurance or plan begins to pay.**
3. What is an out of pocket maximum?
   a. The total amount you pay each year for healthcare premiums.
   b. **The most you pay during a policy period (usually a year) before your health insurance or plan begins to pay 100% of the allowed amount (not including your premium).**
   c. The maximum amount an insurance company will pay for a single medical procedure.
   d. The amount you must pay for a doctor’s visit before your insurance will kick in.
4. Select examples of out of pocket costs:
   a. Prescription medications
   b. Elective procedures
   c. Copay
   d. Deductible
   e. **All of the above**
5. What is a copay?
   a. **A fixed amount (for example, $15) you pay for covered health care services to providers who contract with your health insurance or plan.**
   b. The total amount you pay each year for healthcare premiums.
   c. The total amount your insurance company will pay for your medical expenses in a year.
   d. The percentage of medical costs covered by your insurance after a certain limit is reached.
6. What is an insurance network?
   a. The medical providers your health insurer or plan has contracted with to provide health care services.
   b. The medical facilities your health insurer or plan has contracted with to provide health care services.
   c. The medical suppliers your health insurer or plan has contracted with to provide health care services.
   d. **All of the above.**
7. Which of the following patients qualify for Medicare?
   a. **65+**
   b. **End stage renal disease (any age)**
   c. **Amyotrophic lateral sclerosis (any age)**
   d. Pregnant person
   e. Low income family
8. Which of the following patients qualify for Medicaid?
   a. 65+
   b. End stage renal disease (any age)
   c. Amyotrophic lateral sclerosis (any age)
   d. **Pregnant person**
   e. **Low income family**
9. What type of insurance plan limits coverage to care from doctors who work for or contract with the insurance plan, except in an emergency, and focuses on integrated care and focus on prevention and wellness?
   a. Exclusive Provider Organization (EPO)
   b. **Health Maintenance Organization (HMO)**
   c. Point of Service (POS)
   d. Preferred Provider Organization (PPO)
10. What type of insurance plan allows you to pay less if you use providers in the plan’s network or use doctors, hospitals, and providers outside of the network without a referral for an additional cost?
    a. Exclusive Provider Organization (EPO)
    b. Health Maintenance Organization (HMO)
    c. Point of Service (POS)
    d. **Preferred Provider Organization (PPO)**
11. Which of the following insurance programs are public?
    a. **Medicaid**
    b. **Medicare**
    c. Blue Cross Blue Shield
    d. Cigna
    e. United Healthcare
12. You are working in the Emergency Room and a patient that recently moved to Connecticut is uninsured and is experiencing food insecurity. Which of the following healthcare professionals should you include in the patient’s care to help address this issue?
    a. Occupational Therapist
    b. Physical Therapist
    c. **Social worker**
    d. Nurse
13. Three of the following patients would qualify for Medi**care**. Which one should apply for Medi**caid**?
    a. 42-year-old woman with Type 1 diabetes and end-stage renal disease
    b. 65-year-old widow who was a homemaker for the past 40 years while her husband worked
    c. **20-year-old unemployed single mother who is pregnant with her second child**
    d. 51-year-old man who is disabled due to schizophrenia
14. Which of the following mandates was **NOT** included in the Affordable Care Act (Obamacare)?
    a. **Any student enrolled in higher education can stay on their parent’s insurance plan**
    b. Patients cannot be excluded from insurance coverage due to pre-existing health conditions
    c. Insurance plans must cover preventative services without cost to the patient
    d. Patients who can afford insurance but choose not to buy it have to pay a penalty to the government.

**Self-Efficacy Assessment:** (4 Point Likert scale: Strongly Disagree - Disagree - Agree - Strongly Agree)

Knowledge of Community Resources:

1. I am skilled at identifying barriers to healthcare by taking a patient’s social history.

Strongly Disagree - Disagree - Agree - Strongly Agree

2. I feel comfortable identifying and arranging resources outside of medical treatment that would be beneficial to my patients who are in need.

Strongly Disagree - Disagree - Agree - Strongly Agree

3. I feel confident in identifying the role of other healthcare professionals in addressing patient barriers to care, including insurance and community programs.

Strongly Disagree - Disagree - Agree - Strongly Agree

Knowledge of Health Insurance and Health Systems:

4. I am comfortable explaining deductibles.

Strongly Disagree - Disagree - Agree - Strongly Agree

5. I am comfortable explaining out of pocket costs.

Strongly Disagree - Disagree - Agree - Strongly Agree

6. I can explain the role of physicians in billing and coding.

Strongly Disagree - Disagree - Agree - Strongly Agree

7. I feel confident I can educate patients on the basics of their health insurance.

Strongly Disagree - Disagree - Agree - Strongly Agree

**Knowledge of Barriers to Patient Care Assessment:** (4 Point Likert scale: Strongly Disagree - Disagree - Agree - Strongly Agree)

1. The healthcare system treats people unfairly due to their race or socioeconomic status.

Strongly Disagree - Disagree - Agree - Strongly Agree

2. Patients are in control of most factors that affect their health.

Strongly Disagree - Disagree - Agree - Strongly Agree

3. I have had personal struggles navigating health insurance and health systems.

Strongly Disagree - Disagree - Agree - Strongly Agree
