# Supplementary material for: Teaching Health Insurance and Patient Barriers Through an Interprofessional Escape Room for Preclinical Medical and Health Professional Students
Source: MedEdPORTAL. 2026 Oct 30;22:11647. doi: 10.15766/mep_2374-8265.11647 (PMC13720544; doi:10.15766/mep_2374-8265.11647)
Supplement: Supplementary file 1 [file mep_2374-8265.11647-s001.zip › G. Immediate Postevent Evaluation.docx]

**Appendix G: Immediate Post-Event Evaluation of Escape Room Modality of Learning**

**Purpose:** This survey instrument was designed to capture learner reactions to the escape room modality immediately after participation. It evaluates feasibility, engagement, interprofessional collaboration, and perceived educational value.

**Short Answer Questions:**

1. What did you like about this event?
2. What changes would you make to this event?
3. What would you like to learn more in relation to these topics?

**Please rank the following as it pertains to the event:**

(4 Point Likert scale: Strongly Disagree - Disagree - Agree - Strongly Agree)

1. This event provided me with an opportunity to work with a health profession other than my own.

Strongly Disagree - Disagree - Agree - Strongly Agree

2. I have a better understanding of the expertise of different health professionals.

Strongly Disagree - Disagree - Agree - Strongly Agree

3. I can better incorporate the expertise of others to address social determinants of health.

Strongly Disagree - Disagree - Agree - Strongly Agree

4. This event provided an opportunity for teamwork and critical thinking.

Strongly Disagree - Disagree - Agree - Strongly Agree
